# Clinical Insights and Severity of Enterovirus D68 Respiratory Infections in Vietnamese Children

**DOI:** 10.1093/ofid/ofag102

**Published:** 2026-02-26

**Authors:** Hirono Otomaru, Yurika Kawazoe, Hien Anh Thi Nguyen, Hien Minh Vo, Hoang Huy Le, Michiko Toizumi, Katsumi Mizuta, Duc Anh Dang, Hiroyuki Moriuchi, Lay-Myint Yoshida

**Affiliations:** Department of Pediatric Infectious Diseases, Institute of Tropical Medicine, Nagasaki University, Nagasaki, Japan; Clinical Research Center, Nagasaki University, Nagasaki, Japan; Department of Bacteriology, National Institute of Hygiene and Epidemiology (NIHE), Hanoi, Vietnam; Department of Pediatrics, Khanh Hoa General Hospital, Nha Trang, Vietnam; Department of Bacteriology, National Institute of Hygiene and Epidemiology (NIHE), Hanoi, Vietnam; Department of Pediatric Infectious Diseases, Institute of Tropical Medicine, Nagasaki University, Nagasaki, Japan; Department of Microbiology, Yamagata Prefectural Institute of Public Health, Yamagata, Japan; Department of Bacteriology, National Institute of Hygiene and Epidemiology (NIHE), Hanoi, Vietnam; National Research Center for the Control and Prevention of Infectious Diseases (CCPID), Nagasaki University, Nagasaki, Japan; Department of Pediatric Infectious Diseases, Institute of Tropical Medicine, Nagasaki University, Nagasaki, Japan; Graduate School of Biomedical Sciences, Nagasaki University, Nagasaki, Japan

**Keywords:** child, comorbidity, enterovirus D, pneumonia, respiratory tract infections

## Abstract

**Background:**

Enterovirus D68 (EVD68) causes respiratory disease, yet the risk of severe respiratory disease remains incompletely quantified, especially when stratified by comorbidity status. This study aims to describe the clinical features and risk of severe disease in children with EVD68 compared with those with other Rhinoviruses/Enteroviruses, accounting for comorbidity.

**Methods:**

We analyzed 1100 pediatric EV-positive cases from surveillance in Vietnam (2019–2022), excluding viral coinfections. EVD68 was confirmed by real-time PCR. We examined clinical features, treatments, and comorbidities. Standardization methods estimated risk differences (RDs) and risk ratios (RRs) stratified by (1) general comorbidity, (2) asthma, and (3) either of these. Time-to-recovery in intensive care unit (ICU) was compared using Kaplan–Meier methods.

**Results:**

EVD68 was detected in 55/1100 cases (5.0%). Children with EVD68 more often had wheeze, pneumonia, oxygen therapy, and ICU admission. For wheeze, EVD68 was associated with similar elevated risk with and without general comorbidity (RR 1.40 in both; RD 0.27). Pneumonia risk was likewise elevated with EVD68 (with comorbidity: RR 1.17; RD 0.12; without: RR 1.73; RD 0.14). By asthma status, EVD68 increased wheeze risk in both groups; however, the excess was smaller in asthma (RR 1.10; RD 0.09) than non-asthma (RR 1.43; RD 0.29), consistent with baseline wheeze susceptibility in asthma. Pneumonia risk was similar regardless of asthma status. During ICU stay, wheeze persisted longest in both groups. Time-to-recovery was similar between groups.

**Conclusions:**

EVD68 infection was associated with a higher risk of severe disease than Rhinoviruses/Enteroviruses, independent of documented comorbidity, indicating substantial risk even among previously healthy children.

## Teaser

EVD68 infection was associated with greater severity than RV/EV, with higher risk of wheeze and pneumonia, independent of documented comorbidities; ICU course was similar. Ongoing surveillance is warranted worldwide.

Enterovirus D68 (EVD68; species *Enterovirus deconjunctivitis*), a member of the *Picornaviridae* family, was first identified in 1962 as a causative agent of severe respiratory infections. In 2014, the United States and Canada saw a surge in severe pediatric respiratory illness often accompanied by acute flaccid myelitis (AFM)-like weakness and paralysis [1], with subsequent investigations linking the AFM rise to EVD68 (∼20% of AFM respiratory samples positive) [2] and later reports documenting >2000 EVD68 infections worldwide [3–6]. Further retrospective analysis of past samples revealed that EVD68 detection has increased since the late 2000s [7–9]. Respiratory symptoms associated with EVD68 range from mild to severe, and in severe cases, it can lead to dyspnea, wheezing, pneumonia, and hypoxemia [5, 9]. A meta-analysis has estimated the mortality rate of EVD68 infection to be between 0% and 4.4% [10]. Children with a history of asthma or wheezing experience more severe EVD68 infection. In an epidemiological study in the United States, 59% (338/574) of EVD68-positive cases were admitted to intensive care unit (ICU), and 28% (145/511) required mechanical ventilation [11]. The study indicated that patients with a history of asthma or reactive airway disease were more likely to require intensive care and ventilator support than those without. The reported prevalence of asthma or wheezing among cases with EVD68 has varied across studies [6, 12, 13].

Although EVD68 seroprevalence varies by year of study, it generally increases with age [14, 15]. While seroepidemiological studies on EVD68 using cord blood are limited, a study in Taiwan found over 90% of cord blood samples were seropositive [16]. A meta-analysis reported the lowest seroprevalence in 12-month-old infants, rising to a plateau by age 20–29 [17]. These data suggest frequent childhood infection after waning maternal antibodies. However, epidemiologic patterns remain unclear, likely due to limited diagnostics distinguishing EVD68 from rhinovirus or lack of routine testing [18].

Rhinovirus, another *Picornaviridae* virus, accounts for ∼26% of pediatric respiratory hospitalizations [19]. While the infection rates are similar across asthma status, asthma is associated with more severe and prolonged symptoms [20]. Rhinovirus can infect the lower airways regardless of allergic predisposition [21]. Experimental studies show comparable inflammatory responses in both groups. Yet, only some asthmatics exhibit clinical exacerbations, suggesting heightened airway reactivity [22]. By contrast, the pathophysiologic mechanisms of EVD68 remain unclear. Known receptors include intracellular adhesion molecule 5, located on motor neurons [23], sialic acid [24], and major facilitator superfamily domain-containing protein 6 (MFSD6) [25], but their roles in respiratory disease are not fully understood. In this context, it remains unclear whether EVD68 consistently poses a greater risk of lower respiratory tract illness than rhinovirus, particularly among children with a history of asthma or other comorbidities, or whether the risk is comparable to that of rhinovirus in otherwise healthy children without documented underlying disease.

In 2022, a marked increase in EVD68 cases was observed in Nha Trang, Vietnam. This study had three objectives: First, to characterize the clinical features of EVD68 infections. Second, to evaluate the risk of developing severe respiratory illness associated with EVD68 infection, compared with other enterovirus infections, while adjusting for comorbidity status. Third, to compare the clinical course and management of severe EVD68 cases with those of other enterovirus cases, thereby identifying similarities and differences in disease progression and therapeutic interventions.

## METHODS

### Study Site, Study Population, Study Period, and Enrollment

Nha Trang is a city in Khanh Hoa province, central Vietnam, with an under-five population of 20 174 in 2015 [26]. Acute respiratory infection (ARI) surveillance has been conducted at Khanh Hoa General Hospital. Eligible participants were those presenting with either cough or difficulty breathing. Further details have been described previously [26]. Briefly, all children aged 1 month to 14 years who were hospitalized with ARI were invited to participate in the study. Upon enrollment, written informed consent was obtained from legal guardians. Clinical and demographic information was collected for all enrollees. Clinical data included the respiratory symptoms and general danger signs, such as inability to drink, altered consciousness, convulsions, lethargy, poor sucking, toxic appearance, or irritability. We defined clinical pneumonia according to the modified World Health Organization Integrated Management of Childhood Illnesses (IMCI) algorithm: tachypnea (≥60/min in children under 2 months, ≥50/min at 2–11 months, ≥40/min at 1–5 years, ≥30/min at 6–11 years, and ≥20/min at 12 years or older), or chest indrawing [27, 28]. Hereafter, we refer to this as IMCI pneumonia. A nasopharyngeal swab was obtained from all enrolled children. Children admitted to an ICU at the hospital were also enrolled in the same procedure. During admission to the ICU ward, symptoms and clinical management offered to the children were recorded at least once a day, with multiple visits as needed.

The protocol of this research was approved by the institutional ethical review boards of the National Institute of Hygiene and Epidemiology, Vietnam (IRB-VN 01057), and the Institute of Tropical Medicine, Nagasaki University, Japan (09031837-3).

### Virus Detection

Enteroviruses (EV), including rhinoviruses (RV), were screened with polymerase chain reactions (PCR) [29, 30]. Specimens positive for the screening PCR were further tested by real-time PCR with primers and a probe to detect EVD68 [31]. A TaqMan Fast Virus 1-step Master Mix (Thermo Fisher Scientific, Waltham, MA, USA) and StepOnePlus (Applied Biosystems, Foster City, CA, USA) were used for the assay. Because the primary screening PCR does not distinguish between RV and EV, specimens that tested negative for EVD68 were collectively referred to as “RV/EV” in this study. Although species-level typing was not performed for all screening-positive specimens, partial sequencing of the PCR product was conducted for ICU-derived samples to provide additional context (see Supplementary material). Other common respiratory viruses were also screened, as described in the Supplementary material. All reactions were conducted with positive and negative controls. Samples with viral codetections were excluded from the primary analysis to isolate virus-specific clinical associations. Analyses including codetections were additionally conducted.

### Statistical Analysis

We first described the demographic characteristics of all hospitalized children with ARI. Subsequently, the demographic and clinical characteristics of the RV/EV and EVD68 groups were summarized. Comparisons between the RV/EV and EVD68 groups were performed using the Wilcoxon rank sum test, Pearson's χ^2^ test, or Fisher's exact test, as appropriate. Although multiple outcomes were compared in this exploratory analysis, no adjustment for multiple testing was applied, as the aim was to broadly characterize the differences between the groups and to generate hypotheses for future research. Therefore, *P*-values should be interpreted with caution and considered hypothesis-generating.

To evaluate the impact of EVD68 infection on clinical outcomes and required treatment, we employed a causal inference approach using standardization [32]. Logistic regression models adjusted for age in months, sex, and comorbidities, as defined by International Classification of Diseases, 10th Revision (ICD-10) diagnostic codes. The comorbidity groups were defined as follows: (A) presence of documented comorbidities excluding asthma and excluding ARIs (codes starting with “J’); (B) presence of asthma diagnosis (J45 or J46); and (C) either (A) or (B). The diseases classified in (A) were summarized in Supplementary Table 1. Unspecified diagnoses were classified as no comorbidity. Standardized risk differences (RDs) and risk ratios (RRs) for each outcome were estimated by predicting the counterfactual probabilities of outcome occurrence in a hypothetical population with the same covariate distribution, assuming either EVD68 or RV/EV infection. RD reflects the absolute risk difference; RR, the relative risk. Both metrics convey magnitude and relative impact. To investigate heterogeneity by comorbidity status, we presented stratified RDs and RRs. To account for estimation uncertainty, 95% confidence intervals for all standardized estimates were calculated using 1000 bootstrap replicates. We reviewed discharge diagnoses to identify possible cases of AFM or similar neurological conditions. Given the diagnostic challenges of AFM, we considered a case potentially AFM if any disease known to present with acute flaccid paralysis (AFP) was coded at discharge (see Supplementary material).

Finally, we compared the clinical course between ICU-admitted children with EVD68 and those with RV/EV. This analysis included ICU-admitted children with symptom documentation on at least one day during their ICU stay. For each symptom or treatment, the day following its last recorded observation was defined as the day of recovery. If the symptom or treatment persisted until ICU discharge, the child was censored at that time. Time from ICU admission (time 0) to recovery was analyzed using the Kaplan–Meier method and log-rank test. The analysis included only children with symptoms/treatments present on ICU admission. Day-by-day data prior to ICU admission (eg, during general ward stay) were unavailable and were therefore not included in the analysis.

## RESULTS

### Enrolled Study Participants, Enterovirus Positivity, EVD68 Detection

A total of 4669 children were enrolled. Enterovirus screening, detecting both RV and EV, was performed on 4604 samples available for testing. Among these, 1100 samples were confirmed to be positive. The positive samples were subsequently tested for EVD68 by real-time PCR, and 55 were positive. Samples with viral codetections were excluded from the primary analyses to isolate virus-specific clinical associations. The flow of participant enrollment, enterovirus positivity, and EVD68 detection is illustrated in Supplementary Figure 1.

The demographic characteristics of the overall, EV-positive, and EV-negative children are summarized in Table 1. The median age was 17.0 months (IQR, 7.9–29.5) overall, 17.9 months (IQR, 8.9–30.7) in the EV-positive group, and 16.7 months (IQR, 7.7–29.1) in the EV-negative group. The proportion of children with an asthma diagnosis was 7.4% [95% CI, 5.92–9.11] in the EV-positive group and 2.6% [95% CI, 2.04–3.21] in the EV-negative group. The proportion of children with general comorbidity (non-respiratory illnesses) was 4.6% [95% CI, 3.50–6.10] in the EV-positive group and 7.2% [95% CI, 6.29–8.18] in the EV-negative group. Among EV-positive cases, the median age was 20.9 months (IQR, 11.3–39.0) in the EVD68 group and 17.9 months (IQR, 8.8–30.2) in the RV/EV group (Table 1). In addition, age and asthma distributions were descriptively compared by year of admission. The 95% confidence intervals for asthma prevalence overlapped in all comparisons (Supplementary Table 2).

**Table 1.** Demographic Characteristics of Enrolled Cases

	Enterovirus Infection			EVD68 or RV/EV Infection	
Characteristics	Overall N = 4066	EV-negative N = 2966	EV-positive N = 1100	EVD68 N = 55	RV/EV N = 1045
Year of sampling (n (%), [95% CI])					
2019	1206 (29.7), [28.3–31.1]	925 (31.2), [29.5–32.9]	281 (25.5), [23.0–28.3]	9 (16.4), [8.20–29.3]	272 (26.0), [23.4–28.8]
2020	969 (23.8), [22.5–25.2]	647 (21.8), [20.3–23.4]	322 (29.3), [26.6–32.1]	7 (12.7), [5.69–25.1]	315 (30.1), [27.4–33.0]
2021	754 (18.5), [17.4–19.8]	503 (17.0), [15.6–18.4]	251 (22.8), [20.4–25.4]	1 (1.8), [0.095–11.0]	250 (23.9), [21.4–26.7]
2022	1137 (28.0), [26.6–29.4]	891 (30.0), [28.4–31.7]	246 (22.4), [20.0–25.0]	38 (69.1), [55.0–80.5]	208 (19.9), [17.6–22.5]
Age month (Median (IQR))	17.0 (7.9, 29.5)	16.7 (7.7, 29.1)	17.9 (8.9, 30.7)	20.9 (11.3, 39.0)	17.9 (8.8, 30.2)
Age group (n (%), [95% CI])					
1–5 mo	786 (19.3), [18.1–20.6]	595 (20.1), [18.6–21.6]	191 (17.4), [15.2–19.8]	9 (16.4), [8.20–29.3]	182 (17.4), [15.2–19.9]
6–11 mo	706 (17.4), [16.2–18.6]	520 (17.5), [16.2–19.0]	186 (16.9), [14.8–19.3]	7 (12.7), [5.69–25.1]	179 (17.1), [14.9–19.6]
12–23 mo	1178 (29.0), [27.6–30.4]	849 (28.6), [27.0–30.3]	329 (29.9), [27.2–32.7]	14 (25.5), [15.1–39.3]	315 (30.1), [27.4–33.0]
≥24 mo	1396 (34.3), [32.9–35.8]	1002 (33.8), [32.1–35.5]	394 (35.8), [33.0–38.7]	25 (45.5), [32.2–59.3]	369 (35.3), [32.4–38.3]
Sex (n (%), [95% CI])					
Male	2398 (59.0), [57.4–60.5]	1723 (58.1), [56.3–59.9]	675 (61.4), [58.4–64.2]	33 (60.0), [45.9–72.7]	642 (61.4), [58.4–64.4]
Female	1668 (41.0), [39.5–42.6]	1243 (41.9), [40.1–43.7]	425 (38.6), [35.8–41.6]	22 (40.0), [27.3–54.1]	403 (38.6), [35.6–41.6]
Diagnosis of asthma (n (%)), [95% CI])	157 (3.9), [3.30–4.51]	76 (2.6), [2.04–3.21]	81 (7.4), [5.92–9.11]	7 (12.7), [5.69–25.1]	74 (7.1), [5.64–8.85]
Diagnosis of non-respiratory illness (n (%), [95% CI])	264 (6.5), [5.76–7.31]	213 (7.2), [6.29–8.18]	51 (4.6), [3.50–6.10]	4 (7.3), [2.36–18.4]	47 (4.5), [3.36–5.98]

Column heads are used as denominators.

Abbreviations: EV, enterovirus; EVD68, enterovirus D68; RV/EV, rhinoviruses/enteroviruses; CI, confidence interval; IQR, interquartile range.

### Seasonality of EVD68 and RV/EV

**Figure 1.**
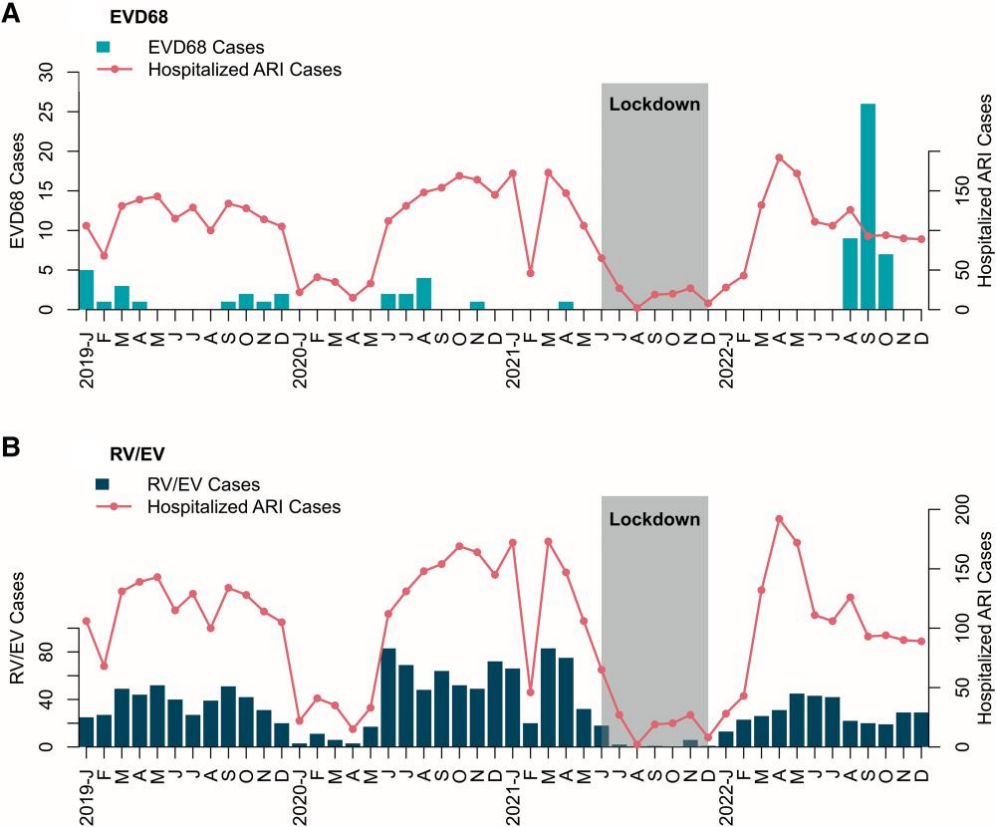
Epidemic curves of hospitalized acute respiratory infection (ARI) cases with EVD68 (*A*) and RV/EV (*B*). Bars represent case counts for EVD68 and RV/EV, respectively. The solid line indicates the total number of hospitalized ARI cases over time. The x-axis shows month/year and the y-axis shows case counts. EVD68 activity concentrates in 2022, whereas RV/EV is observed across the period.

Figure 1 presents the monthly case numbers for all cases and for children with EVD68 or RV/EV. Social distancing measures and movement restrictions related to COVID-19, implemented from April 2020 onward and more stringently from June to December 2021, were associated with a marked decline in registered cases. EVD68 was detected from January to April and September to December 2019, June to November 2020, April 2021, and August to October 2022. These detections occurred at irregular intervals, suggesting nonseasonal but recurrent circulation. No clear association with seasonal climate patterns, such as the rainy season, was observed. A pronounced increase in children with EVD68 was observed between August and October 2022, accounting for 62% of the study's EVD68 detections. In contrast, children with RV/EV were identified almost continuously throughout the surveillance period except during the period of COVID-19 restrictions.

### Clinical Symptoms and Required Treatment

Next, the clinical symptoms and treatments administered were compared between the EVD68 and RV/EV groups (Table 2). The median age was not significantly different between the groups. The diagnosis of asthma or reported general comorbidities were slightly common in the EVD68 group. No cases of AFM or related neurologic conditions were identified in either group based on discharge diagnoses. Children in the EVD68 group more frequently had chest indrawing (34.5%), abnormal lung sounds (such as stridor (52.7%) or crackles (61.8%)), wheezing (96.4%), IMCI pneumonia (36.4%), and danger signs (12.7%). In blood test results, the EVD68 group exhibited higher neutrophil (53.6%) and eosinophil (1.6%) counts in the white blood cell differential. The data of lymphocytes is unavailable. The median length of hospital stay was longer in the EVD68 group (6.0 days), while the time from symptom onset to discharge showed no notable difference. Regarding treatment, ICU admission (20%), oxygen supplementation (18%), corticosteroid use (73%), and β₂-agonist inhalation (67%) were administered more frequently in the EVD68 group. Mechanical ventilation was slightly more common in the EVD68 group. Other measures did not differ between groups. Analyses including cases with viral codetections showed broadly similar results to those of the primary analyses (Supplementary Tables 3 and 4).

**Table 2.** Clinical Symptoms and Required Treatment

Group	Characteristics	EVD68 N = 55	RV/EV N = 1045	*P* Value
Demographics	Age month (median (IQR))	20.9 (11.3, 39.0)	17.9 (8.8, 30.2)	.3^a^
	Age group (no (%))	…	…	.5^b^
	1–5 mo	9 (16.4%)	182 (17.4%)	…
	6–11 mo	7 (12.7%)	179 (17.1%)	…
	12–23 mo	14 (25.5%)	315 (30.1%)	…
	≥24 mo	25 (45.5%)	369 (35.3%)	…
Symptoms	Cough (no. (%))	55 (100.0%)	1035 (99.0%)	>.9^c^
	Difficulty in breathing (no. (%))	29 (52.7%)	428 (41.0%)	.084^b^
	Tachypnea (no. (%))	13 (23.6%)	149 (14.3%)	.056^b^
	Body temperature (median (IQR))	37.5 (37.0, 38.5)	37.8 (37.0, 38.5)	.2^a^
	Chest indrawing (no. (%))	19 (34.5%)	166 (15.9%)	<.001^b^
	Stridor (no. (%))	29 (52.7%)	316 (30.2%)	<.001^b^
	Wheeze (no. (%))	53 (96.4%)	721 (69.0%)	<.001^b^
	Crackle (no. (%))	34 (61.8%)	377 (36.1%)	<.001^b^
	IMCI pneumonia (no. (%))	20 (36.4%)	222 (21.2%)	.008^b^
	Presence of danger sign (no. (%))	7 (12.7%)	45 (4.3%)	.012^c^
Comorbidity	Diagnosis of asthma (no. (%))	7 (12.7%)	74 (7.1%)	.12^c^
	Diagnosis of general comorbidity (no. (%))	4 (7.3%)	47 (4.5%)	.3^c^
Blood Testing	RBC (cells/µl) (median (IQR))	4.5 (4.1, 5.0)	4.4 (4.1, 4.7)	.055^a^
	Platelet (/µl) (cells/µl) (median (IQR))	368.0 (283.0, 463.0)	344.0 (274.0, 421.0)	.10^a^
	WBC (cells/µl) (median (IQR))	12.6 (9.4, 17.9)	12.0 (9.3, 15.2)	.2^a^
	WBC differential (% neutrophils) (median (IQR))	53.6 (38.0, 66.5)	46.0 (32.1, 61.3)	.040^a^
	WBC differential (% eosinophils) (median (IQR))	1.6 (0.4, 3.3)	1.0 (0.2, 2.4)	.033^a^
	WBC differential (% monocytes) (median (IQR))	9.3 (6.2, 11.7)	9.1 (7.0, 12.0)	.3^a^
Treatment	Duration of hospitalization (days, median (IQR))	6.0 (4.0, 8.0)	5.0 (3.0, 7.0)	.008^a^
	Duration of onset to discharge (days, median (IQR))	8.0 (6.0, 12.0)	8.0 (5.0, 11.0)	.13^a^
	ICU admission (no. (%))	11 (20%)	104 (10.0%)	.018^b^
	Oxygen (no. (%))	10 (18%)	82 (7.8%)	.020^c^
	Mechanical ventilation (no. (%))	2 (3.6%)	9 (0.9%)	.10^c^
	Steroid (no. (%))	40 (73%)	508 (49%)	<.001^b^
	Beta2-agonists (inhaled) (no. (%))	31 (67%)	378 (50%)	.021^b^
	Unknown	9	287	…
	Beta2-agonists (oral) (no. (%))	0 (0%)	0 (0%)	>.9^c^
	Unknown	9	287	…

Column heads are used as denominators.

Treatment variables often occur in linked clinical pathways; therefore, they are presented for descriptive context only and are not interpreted as independent outcomes.

Abbreviations: EVD68, Enterovirus D68; RV/EV, rhinoviruses/enteroviruses; RBC, red blood cell; WBC, white blood cell; ICU, intensive care unit; IQR, interquartile range; IMCI, integrated management of childhood illnesses.

^a^Wilcoxon rank sum test.

^b^Pearson's χ^2^ test.

^c^Fisher's exact test.

### Association of EVD68 Infection With Clinical Pneumonia and Wheezing

**Figure 2.**
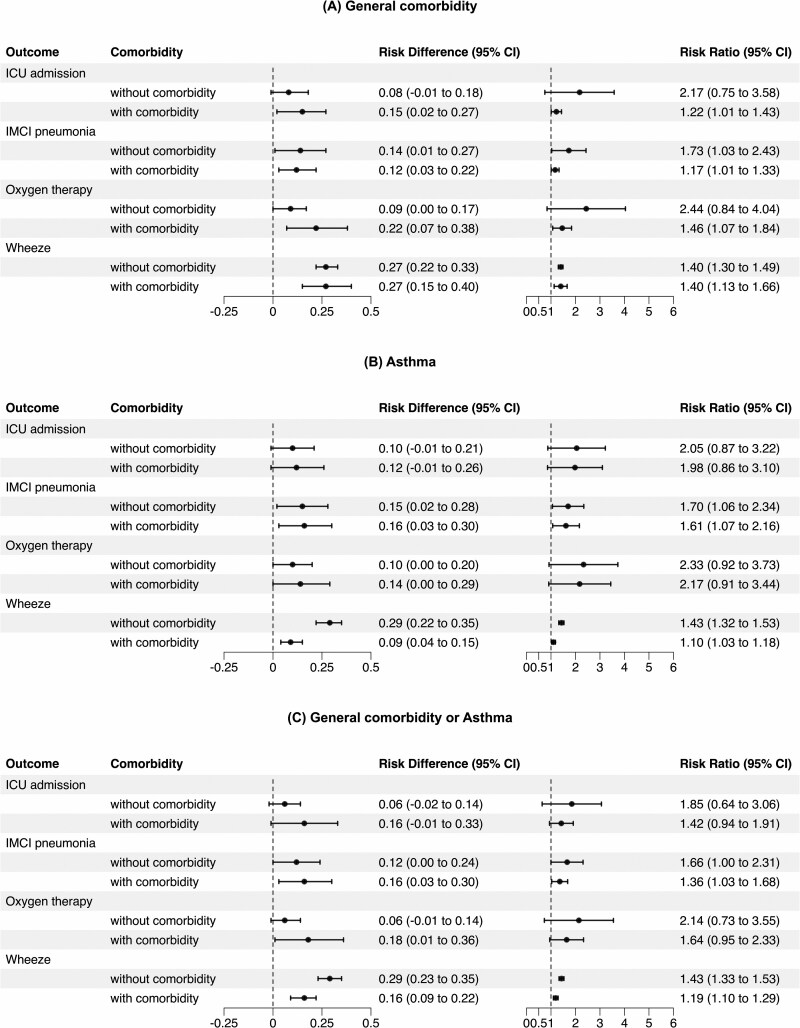
Adjusted risk estimates for clinical outcomes associated with Enterovirus D68 (EVD68) infection, stratified by comorbidity status. Risk differences (RD) and risk ratios (RR) for IMCI pneumonia, wheezing, oxygen therapy, and ICU admission comparing EVD68-positive and RV/EV-positive cases, (*A*) stratified by the presence of general comorbidities (excluding asthma and acute respiratory infections), (*B*) stratified by asthma diagnosis, and (*C*) stratified by the presence of either (*A*) or (*B*) comorbidities. Wheeze and IMCI pneumonia were elevated across strata.

We examined the effect of EVD68 infection on clinical outcomes (IMCI pneumonia, wheezing, ICU admission, and oxygen therapy) using a standardization-based causal inference approach, stratified by comorbidity status. Analyses considered: (A) general comorbidities excluding asthma; (B) asthma; and (C) either condition. Results are shown in Figure 2*A*–*C*.

Overall, children with EVD68 infection exhibited a trend toward increased relative risks and RDs compared with those with RV/EV infection. Compared with RV/EV infection, EVD68 infection showed similarly elevated RRs of wheezing in children with and without general comorbidities (RR: 1.40 [95% CI: 1.13–1.66] vs 1.40 [1.30–1.49]), with the RDs of 0.27 in both groups (RD: 0.27 [0.15–0.40] vs 0.27 [0.22–0.33]) (Figure 2*A*). For IMCI pneumonia, the RR was similar in children with general comorbidities and those without (RR: 1.17 [1.01–1.33] vs 1.73 [1.03–2.43]), while the RD was similar (RD: 0.12 [0.03–0.22] vs 0.14 [0.01–0.27]). Oxygen therapy was more common in EVD68 cases with comorbidities. (RD: 0.22 [0.07–0.38]; RR: 1.46 [1.07–1.84]), whereas the results among those without comorbidities were inconclusive, as the lower bounds of the 95% confidence intervals did not exceed the null value.

In Figure 2*B*, we observed an effect modification by asthma status on the association between EVD68 infection and wheezing. While EVD68 infection increased the RR of wheezing in both children with asthma (1.10 [1.03–1.18]) and those without asthma (1.43 [1.32–1.53]), the magnitude of this increase was smaller in children with asthma. The RD was also lower in children with asthma (RD: 0.09 [0.04–0.15]) compared with those without asthma (RD: 0.29 [0.22–0.35]). In contrast, the RR and RD for IMCI pneumonia were similarly elevated with EVD68 infection regardless of asthma status. We performed stratified analyses by admission year (2022 vs non-2022), adjusting for age, sex, and asthma status; however, no significant year-specific heterogeneity was observed in the association between EVD68 and the main outcomes (see Supplementary material).

Similar trends of effect modification were observed in Figure 2*C*. EVD68 infection was associated with an increased risk of wheezing in both children with (RR: 1.19 [1.10–1.29]; RD: 0.16 [0.09–0.22]) and without (RR: 1.43 [1.33–1.53]; RD: 0.29 [0.23–0.35]) comorbidities. However, the magnitude of the association was attenuated among children with comorbidities. A similar pattern was observed for IMCI pneumonia, although the confidence interval for the RD in children without comorbidities crossed the null.

### Clinical Progression After ICU Admission

**Figure 3.**
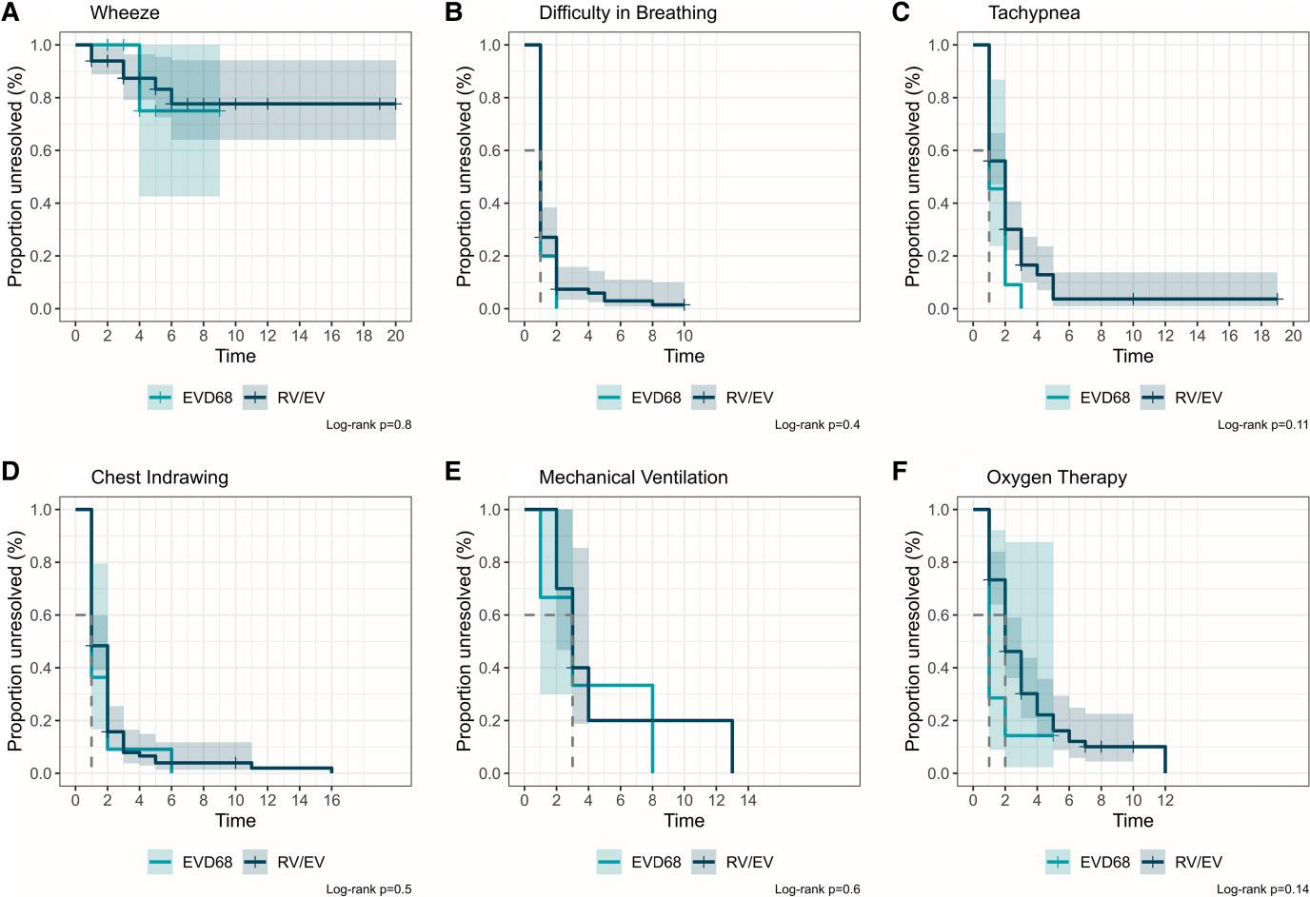
Kaplan–Meier plots of clinical recovery among intensive care unit (ICU)-admitted cases with specific symptoms or treatments during ICU stay. Plots show time from ICU admission to recovery for patients who exhibited each symptom or received each treatment on the day of ICU admission. Panels show resolution of: (*A*) wheeze (n = 11, n = 82, EVD68 or RV/EV, respectively), (*B*) difficulty in breathing (n = 10, n = 85), (*C*) tachypnea (n = 11, n = 100), (*D*) chest indrawing (n = 11, n = 91), (*E*) mechanical ventilation (n = 3, n = 10), and (*F*) oxygen therapy (n = 7, n = 75). Discharge before recovery was treated as a censoring event. The x-axis represents time in days from ICU admission. The y-axis represents the proportion of participants with unresolved symptoms or ongoing treatment. The shaded areas represent the 95% confidence intervals. Trajectories were broadly similar between groups.

To assess clinical progression after ICU admission, we used Kaplan–Meier curves to compare time-to-recovery of symptoms and discontinuation of treatments between children with EVD68 and those with RV/EV (Figure 3). Individual ICU trajectories from admission to discharge are shown for context (Supplementary Figure 2). Among all groups, wheezing was the most persistent symptom. No significant differences in time-to-recovery were observed across groups based on the log-rank test.

## DISCUSSION

We investigated clinical and epidemiological features of EVD68 among hospitalized children with ARIs in Nha Trang, Vietnam. Children with EVD68 more frequently had wheeze, IMCI pneumonia, received oxygen therapy and were admitted to ICU compared with RV/EV. The associations with wheeze and IMCI pneumonia were observed regardless of comorbidity status. Although children with EVD68 increased in 2022, admission year–stratified analyses, adjusted for age, sex, and asthma status, showed no significant heterogeneity. ICU recovery patterns were generally similar between EVD68 and RV/EV groups. These findings emphasize distinct features of EVD68 and its potential for severe illness.

Previous studies have shown that EVD68 tends to circulate in autumn in temperate regions [7, 33–37]. In the United States, major outbreaks have occurred every 2 years (eg, 2014, 2018, and 2022), with smaller peaks in between [33, 34, 38]. Similarly, in Japan, outbreaks were reported in 2005, 2010, 2013, 2015, 2018, and 2022 [7, 5, 39, 40], suggesting a multiyear cycle rather than strictly annual or biennial patterns. In contrast, our data showed irregular detection pattern, suggesting a circulation pattern that differs from those observed in temperate regions. This irregularity may be influenced by viral introductions from both hemispheres, evidenced by detections in both rainy and dry seasons without clear seasonality or rainfall association. In contrast to EVD68, RV/EV were detected throughout most of the year, consistent with previous findings in tropical settings [41, 42].

As reported elsewhere [18, 38, 43], strict behavioral restrictions during the SARS-CoV-2 pandemic likely curtailed EVD68 transmission, thereby reducing population-level immunity. An immunoepidemiological study reported high EVD68 exposure among previously seronegative children after lifting non-pharmaceutical interventions, consistent with pandemic-related immunity gaps [44]. Decreased exposure during lockdowns may have left children with insufficient immunological protection, increasing their susceptibility and hospitalization risk upon re-exposure. Although the introduction of a novel variant cannot be excluded, genomic analyses in United States showed that 2022 strains belonged to the B3 subclade and were closely related to 2018 strains [38]. Molecular epidemiologic investigations, including seroepidemiology and genomic surveillance, are warranted to clarify EVD68 transmission dynamics, assess population immunity, and explore potential variant introductions in Vietnam and Southeast Asia.

Children with EVD68 infection exhibited higher frequencies of wheezing and IMCI pneumonia than those with RV/EV. We assessed the association between EVD68 infection and severe respiratory outcomes, adjusting for comorbidities. While previous studies emphasized asthma or reactive airway disease, our study covered broader comorbidities and showed that even children without documented comorbidities had increased risks of wheezing and IMCI pneumonia. These findings suggest that prior comorbidities may not be necessary prerequisites for EVD68-associated lower respiratory tract complications, and that previously healthy children can also be at substantial risk. Consistent with this, a larger study of hospitalized children with EVD68-positive ARI reported severe disease even among otherwise healthy children [45].

Interestingly, the magnitude of this increased risk of wheeze was less pronounced in children with asthma than in children without asthma. This effect modification may be due to children with asthma being prone to wheeze with both viruses, whereas in children without asthma, wheezing may be more specifically attributable to EVD68. A modest eosinophil elevation in children with EVD68 may indicate an interaction with allergic predisposition. An animal study support this, showing allergic sensitization can amplify EVD68-induced inflammation [46]. However, the underdiagnosis of allergic or chronic diseases may have led to underestimation of these associations. As most EVD68 infections are likely subclinical and go unrecognized in early childhood, improving case detection and integrating clinical and immunological profiling could enhance our understanding of risk factors for severe disease. Continued differentiation of EVD68 from other enteroviruses and sustained surveillance will be essential for understanding its epidemiology and pathophysiology.

Although AFM has been linked to EVD68 in prior outbreaks, no AFP/AFM was observed in this study. This may reflect circulation of less neurotropic strains, consistent with reports noting no AFM increase despite EVD68 surges [47], and/or limited ascertainment in an ARI-based surveillance system without systematic neurologic assessment. Neurologic presentations without respiratory symptoms, or subtle signs in infants, may have been missed. However, without systematic data collection, this remains unconfirmed.

Among children admitted to ICU, we found no clear differences in recovery time from respiratory symptoms between the EVD68 and RV/EV groups. Although EVD68 infection may predispose to severe illness, the clinical course post-ICU admission appears similar. Prior studies comparing ICU trajectories between EVD68 and other enteroviruses are limited but show comparable outcomes [48]. While the infected virus may influence recovery time, such differences were small and undetectable due to limited statistical power. Further investigations involving larger cohorts are warranted to better characterize the recovery patterns.

This study has several limitations. First, data prior to ICU admission were not systematically captured in this study, possibly underestimating total illness duration. However, since ICU discharge typically follows the resolution of critical conditions such as the need for oxygen therapy, it is reasonable to infer that recovery from the most severe phase of illness likely occurred during ICU stay. Second, comorbidity information based on ICD-10 codes was not validated against medical records, and some misclassification cannot be excluded. Although asthma diagnoses were less frequently assigned in younger children, this age-dependent pattern was similar across groups and age was adjusted for in the analyses; however, small numbers within comorbidity subcategories precluded further stratified analyses, and residual confounding due to unmeasured factors may remain. Third, our inclusion criteria preferentially captured lower respiratory tract infections; thus, findings primarily apply to ARI. Although enteroviruses have diverse clinical presentations, multicountry European surveillance has shown that EVD68 is strongly associated with respiratory samples, supporting respiratory disease as its predominant clinical phenotype [49]. Fourth, molecular typing was conducted only for ICU cases, among whom the typed RV/EV subgroup consisted predominantly of rhinoviruses. For non-ICU cases, routine typing was not performed, and residual sample volumes were insufficient for sequencing, precluding finer differentiation within the RV/EV group. Lastly, radiographic findings and inflammatory markers were unavailable; therefore, disease severity was assessed using clinical symptoms and care-utilization outcomes.

## CONCLUSIONS

In summary, this study compared the epidemiological and clinical characteristics of EVD68 and other RV/EV infections in hospitalized children in Nha Trang, Vietnam. We found an increased risk of clinical pneumonia and wheezing among children with EVD68 infection, irrespective of comorbidity status. Clinical progression in ICU was comparable between groups. These findings provide additional insight into EVD68-associated disease severity and underscore the importance of continued research to guide clinical care and public health responses to EVD68 outbreaks.

## Supplementary Material

ofag102_Supplementary_Data

## Supplementary Data

Supplementary materials are available at *Open Forum Infectious Diseases* online. Consisting of data provided by the authors to benefit the reader, the posted materials are not copyedited and are the sole responsibility of the authors, so questions or comments should be addressed to the corresponding author.

## Notes

***Author Contributions*.** Conceptualization: H.O.; Methodology: H.O. and Y.K.; Formal analysis: H.O. and Y.K.; Investigation: H.N. and H.V.; Data curation: H.L.; Visualization: H.O.; Writing—original draft: H.O.; Writing—review & editing: H.O. and M.T.; Supervision: M.T., K.M., H.M., D.D., and L.Y.; Funding acquisition: H.O. and L.Y.

***Acknowledgments*.** We thank the study participants and their parents, as well as the staff of Khanh Hoa General Hospital and Khanh Hoa Health Service, for their support of the study. We also thank the laboratory technician who assisted with sample testing at the Department of Pediatric Infectious Diseases, Institute of Tropical Medicine, Nagasaki University.

***Data availability*.** Deidentified individual-level data underlying the results are available from the corresponding author upon reasonable request, subject to a data-use agreement and, where required, approval by the relevant ethics committees. Analysis code and variable definitions are available on request.

***Patient consent statement*.** Written informed consent was obtained from the parents or legal guardians of all enrolled children at the time of enrollment. The study protocol was approved by the Institutional Review Boards of the National Institute of Hygiene and Epidemiology, Vietnam (IRB-VN 01057), and the Institute of Tropical Medicine, Nagasaki University, Japan (09031837-3).

***Financial support*.** This work was supported by the Japan Agency for Medical Research and Development (AMED) under the Japan Program for Infectious Diseases Research and Infrastructure (Grant Number JP21wm0125006 to L.Y.), and the Japan Society for the Promotion of Science Grant-in-Aid for Scientific Research (C) (grant number JP24K13413 to H.O.).
