# Supplementary material for: Clinical Insights and Severity of Enterovirus D68 Respiratory Infections in Vietnamese Children
Source: Open Forum Infect Dis. 2026 Feb 26;13(3):ofag102. doi: 10.1093/ofid/ofag102 (PMC12968390; doi:10.1093/ofid/ofag102)
Supplement: ofag102_Supplementary_Data [file ofag102_supplementary_data.pdf]

Supplementary Material

Title: Clinical insights and severity of Enterovirus D68 respiratory infections in Vietnamese children.

Authors: Hirono Otomaru; Yurika Kawazoe; Hien Anh Thi Nguyen; Hien Minh Vo; Hoang Huy Le; Michiko Toizumi; Katsumi Mizuta; Duc Anh Dang; Hiroyuki Moriuchi; Lay-Myint Yoshida

1. Virus detection

We extracted RNA from nasopharyngeal swabs with a QIAGEN Viral RNA mini kit (QIAGEN Inc., Valencia, CA, USA). Respiratory viruses, including the influenza virus, respiratory syncytial virus, parainfluenza virus, adenovirus, bocavirus, and coronaviruses (229E and OC43), were screened using multiplex polymerase chain reactions (PCR). A screening for coronaviruses (NL63, HKU1, and SARS-CoV-2) was conducted for the samples collected after March 2021. Enterovirus was screened with another PCR [1,2]. Electrophoresis with a 2% agarose gel was performed to confirm the amplification. Detailed information, including reaction conditions and protocol, has been described previously [3].

2. Nucleotide sequencing of screening PCR product

For ICU cases, enterovirus PCR-positive specimens were further examined by nucleotide sequencing, as the screening assay targets both enteroviruses (EV) and rhinoviruses (RV). The PCR product, targeting the 5′ untranslated region (5′UTR) of enterovirus, was purified using ExoSAP-IT Express (Thermo Fisher Scientific, Waltham, MA, USA). Sequencing reactions were performed using the BigDye Terminator v3.1 Cycle Sequencing Kit and run on a 3730XL DNA Analyzer (Applied Biosystems, Foster City, CA, USA). Sequence similarities were assessed using BLAST against the GenBank database. Among the ICU samples that underwent 5′UTR sequencing (n=115), most showed highest similarity to rhinoviruses (n=95, 82.6%). The remaining samples were classified as EVD68 (n=7), other enteroviruses (n=4), or were unclassifiable due to insufficient read quality (n=9). Given the limited discriminatory power of this region, these findings should be interpreted with caution. Consequently, throughout this study, specimens that tested positive in the screening PCR but negative for EVD68 were categorized as RV/EV.

3. Discharge diagnosis analysis

We also reviewed discharge diagnoses of children with either RV/EV or EVD68 to identify possible cases of AFM or similar neurological conditions. Given the diagnostic challenges of AFM, we considered a case potentially AFM if any disease known to present with acute flaccid paralysis (AFP) was coded at discharge. According to AFP surveillance guidelines [4], such

diseases include poliomyelitis-like paralysis, A80 (Acute poliomyelitis), G04.0 (Acute disseminated encephalomyelitis), G04.8 (Other encephalitis, myelitis, and encephalomyelitis), G04.9 (Encephalitis, myelitis, and encephalomyelitis, unspecified), G37.3 (Acute transverse myelitis in demyelinating disease of central nervous system), and G61.0 (Guillain-Barré syndrome).

4. Sensitivity analysis stratified by admission year

Because cases with EVD68 were concentrated in 2022, we conducted stratified analyses by admission year (2022 vs. non-2022) to assess potential single-year effects. For the main outcomes (IMCI pneumonia, wheezing, ICU admission, and oxygen supplementation), risk differences and risk ratios were estimated separately by year using the same standardization approach, adjusted for age in months, sex, and asthma status, as in the primary analysis. Effect heterogeneity by year was evaluated using bootstrap-based p-values for heterogeneity (p-heterogeneity). The results are presented in Supplementary Table 5. A non-significant p-heterogeneity indicates no evidence that the association between EVD68 and outcomes differed by admission year.

Supplementary Tables

Supplementary Table 1. Detailed Classification of General Comorbidities.

Category	EVD68 Group	RV/EV Group
**[General comorbidity, yes)]***	**4**	**47**
Congenital Disorders (Q)	2	12
Neurological Disorders (G)	3	5
Nutritional/Metabolic Disorders (E)	0	3
Tuberculosis (A15-A19)	0	5
Other Comorbidities **	0	23

* The total count is not the sum of the individual categories shown in the table due to the overlap of comorbidities (i.e., a single patient may have multiple diagnoses).

** Other comorbidities include chronic diseases (Hematologic, Malignancy or other diseases), complications, or non-respiratory infections.

Supplemental Table 2: Demographic characteristics of children with EVD68 and RV/EV infection, stratified by admission year (2022 vs. non-2022).

Section	Item	Subitem	2022	non-2022
EVD68	Number of cases		38	17
	Age, median (IQR)		19.4 (10.4–42.5)	25.9 (12.3–35.6)
	Age group in months, n (% [95%CI])	0-5	6 (15.8% [6.0–31.3])	3 (17.6% [3.8–43.4])
		6-11	6 (15.8% [6.0–31.3])	1 (5.9% [0.1–28.7])
		12-23	10 (26.3% [13.4–43.1])	4 (23.5% [6.8–49.9])
		≧24	16 (42.1% [26.3–59.2])	9 (52.9% [27.8–77.0])
	Asthma, n (% [95%CI])		3 (7.9% [1.7–21.4])	4 (23.5% [6.8–49.9])
	Asthma Age group in months, n (% [95%CI])	0-5	0 (0.0% [0.0–9.3])	0 (0.0% [0.0–19.5])
		6-11	1 (2.6% [0.1–13.8])	0 (0.0% [0.0–19.5])
		12-23	1 (2.6% [0.1–13.8])	1 (5.9% [0.1–28.7])
		≧24	1 (2.6% [0.1–13.8])	3 (17.6% [3.8–43.4])
RV/EV	Number of cases		208	837
	Age (median, IQR)		19.2 (11.4–29.5)	17.3 (7.8–30.7)
	Age group (months) , n (% [95%CI])	0-5	23 (11.1% [7.1–16.1])	159 (19.0% [16.4–21.8])
		6-11	32 (15.4% [10.8–21.0])	147 (17.6% [15.0–20.3])
		12-23	78 (37.5% [30.9–44.5])	237 (28.3% [25.3–31.5])
		≧24	75 (36.1% [29.5–43.0])	294 (35.1% [31.9–38.5])
	Asthma, n (% [95%CI])		15 (7.2% [4.1–11.6])	59 (7.0% [5.4–9.0])
	Asthma Age group (months) , n (% [95%CI])	0-5	0 (0.0% [0.0–1.8])	1 (0.1% [0.0–0.7])
		6-11	1 (0.5% [0.0–2.6])	3 (0.4% [0.1–1.0])
		12-23	3 (1.4% [0.3–4.2])	14 (1.7% [0.9–2.8])
		≧24	11 (5.3% [2.7–9.3])	41 (4.9% [3.5–6.6])

Supplementary Table 3. Demographic characteristics of enrolled cases, including those with viral co-detections. Column header counts are used as denominators.

	Enterovirus infection			EVD68 or RV/EV infection	
**Characteristics**	**Overall**<br>N = 4604	**EV-negative**<br>N = 2966	**EV-positive**<br>N = 1638	**EVD68**<br>N = 68	**RV/EV**<br>N = 1570
**Year of Sampling (n (%), [95% CI])**					
2019	1,388 (30.1), [28.8–31.5]	925 (31.2), [29.5–32.9]	463 (28.3), [26.1–30.5]	16 (23.5), [14.4–35.6]	447 (28.5), [26.3–30.8]
2020	1,133 (24.6), [23.4–25.9]	647 (21.8), [20.3–23.4]	486 (29.7), [27.5–32.0]	9 (13.2), [6.60–24.1]	477 (30.4), [28.1–32.7]
2021	808 (17.5), [16.5–18.7]	503 (17.0), [15.6–18.4]	305 (18.6), [16.8–20.6]	1 (1.5), [0.077–9.01]	304 (19.4), [17.5–21.4]
2022	1,275 (27.7), [26.4–29.0]	891 (30.0), [28.4–31.7]	384 (23.4), [21.4–25.6]	42 (61.8), [49.1–73.0]	342 (21.8), [19.8–23.9]
**Age Month (Median (IQR))**	16.9 (7.9, 28.8)	16.7 (7.7, 29.1)	17.4 (8.3, 28.7)	18.1 (10.8, 37.0)	17.3 (8.2, 28.4)
**Age Group (n (%), [95% CI])**					
1-5mo	886 (19.2), [18.1–20.4]	595 (20.1), [18.6–21.6]	291 (17.8), [16.0–19.7]	10 (14.7), [7.65–25.8]	281 (17.9), [16.1–19.9]
6-11mo	800 (17.4), [16.3–18.5]	520 (17.5), [16.2–19.0]	280 (17.1), [15.3–19.0]	10 (14.7), [7.65–25.8]	270 (17.2), [15.4–19.2]
12-23mo	1,378 (29.9), [28.6–31.3]	849 (28.6), [27.0–30.3]	529 (32.3), [30.0–34.6]	19 (27.9), [18.1–40.3]	510 (32.5), [30.2–34.9]
≧24mo	1,540 (33.4), [32.1–34.8]	1,002 (33.8), [32.1–35.5]	538 (32.8), [30.6–35.2]	29 (42.6), [30.9–55.2]	509 (32.4), [30.1–34.8]
**Sex (n (%), [95% CI])**					
Male	2,727 (59.2), [57.8–60.7]	1,723 (58.1), [56.3–59.9]	1,004 (61.3), [58.9–63.7]	41 (60.3), [47.7–71.7]	963 (61.3), [58.9–63.7]
Female	1,877 (40.8), [39.3–42.2]	1,243 (41.9), [40.1–43.7]	634 (38.7), [36.3–41.1]	27 (39.7), [28.3–52.3]	607 (38.7), [36.3–41.1]
**Diagnosis of Asthma (n (%)), [95% CI])**	173 (3.8), [3.24–4.36]	76 (2.6), [2.04–3.21]	97 (5.9), [4.85–7.20]	7 (10.3), [4.59–20.7]	90 (5.7), [4.66–7.03]
**Diagnosis of Non-respiratory Illness (n (%), [95% CI])**	278 (6.0), [5.38–6.78]	213 (7.2), [6.29–8.18]	65 (4.0), [3.10–5.06]	4 (5.9), [1.90–15.1]	61 (3.9), [3.01–4.99]

Abbreviations: CI, Confidence Interval; IQR, Interquartile Range.

Supplementary Table 4. Clinical symptoms and treatments among cases, including those with viral co-detections. Column header counts are used as denominators.

		**EVD68 Infection Status**		
**Group**	**Characteristics**	**EVD68** N = 68	**RV/EV** N = 1,570	**p-value**
Demographics	Age Month (Median (IQR))	18.1 (10.8, 37.0)	17.3 (8.2, 28.4)	0.2[1]
	Age Group (n (%))			0.4[2]
	1-5mo	10 (14.7%)	281 (17.9%)	
	6-11mo	10 (14.7%)	270 (17.2%)	
	12-23mo	19 (27.9%)	510 (32.5%)	
	≧24mo	29 (42.6%)	509 (32.4%)	
Symptoms	Cough (n (%))	68 (100.0%)	1,557 (99.2%)	>0.9[3]
	Difficulty in Breathing (n (%))	33 (48.5%)	638 (40.6%)	0.2[2]
	Tachypnea (n (%))	16 (23.5%)	237 (15.1%)	0.060[2]
	Body Temperature (Median (IQR))	37.5 (37.0, 38.4)	38.0 (37.0, 38.5)	0.067[1]
	Chest Indrawing (n (%))	21 (30.9%)	258 (16.4%)	0.002[2]
	Stridor (n (%))	36 (52.9%)	503 (32.0%)	<0.001[2]
	Wheeze (n (%))	63 (92.6%)	1,127 (71.8%)	<0.001[2]
	Crackle (n (%))	43 (63.2%)	610 (38.9%)	<0.001[2]
	IMCI Pneumonia (n (%))	23 (33.8%)	354 (22.5%)	0.031[2]
	Presence of Danger Sign (n (%))	8 (11.8%)	74 (4.7%)	0.018[3]
Comorbidity	Diagnosis of Asthma (n (%))	7 (10.3%)	90 (5.7%)	0.12[3]
	Diagnosis of Non-respiratory Illness (n (%))	4 (5.9%)	61 (3.9%)	0.3[3]
Blood Testing	RBC (cells/ul)(Median (IQR))	4.5 (4.2, 4.9)	4.4 (4.1, 4.7)	0.023[1]
	Platelet (/ul)(cells/ul)(Median (IQR))	364.5 (290.0, 467.5)	343.0 (271.0, 424.0)	0.059[1]
	WBC (cells/ul)(Median (IQR))	12.8 (9.2, 17.8)	11.9 (8.9, 15.2)	0.15[1]
	WBC Differential (% Neutrophils)(Median (IQR))	51.4 (38.0, 62.0)	44.5 (30.8, 59.3)	0.038[1]
	WBC Differential (% Eosinophils)(Median (IQR))	1.5 (0.4, 3.4)	0.8 (0.2, 2.2)	0.007[1]
	WBC Differential (% Monocytes)(Median (IQR))	9.1 (6.5, 11.7)	9.6 (7.2, 12.5)	0.12[1]
Treatment	Duration of Hospitalization (days, Median (IQR))	6.0 (4.0, 7.0)	5.0 (3.0, 7.0)	0.017[1]
	Duration of Onset to Discharge (days, Median (IQR))	9.0 (6.0, 11.5)	8.0 (5.0, 11.0)	0.13[1]
	ICU Admission (n (%))	12 (18%)	160 (10%)	0.050[2]
	Oxygen (n (%))	11 (16%)	124 (7.9%)	0.015[2]
	Mechanical Ventilation (n (%))	2 (2.9%)	15 (1.0%)	0.2[3]
	Steroid (n (%))	51 (75%)	774 (49%)	<0.001[2]
	Beta2-Agonists (Inhaled) (n (%))	37 (71%)	573 (52%)	0.007[2]
	Unknown	16	467	
	Beta2-Agonists (Oral) (n (%))	0 (0%)	1 (<0.1%)	>0.9[3]
	Unknown	16	467	

1.Wilcoxon rank sum test, 2.Pearson's Chi-squared test, 3.Fisher's exact test.

Abbreviations: RBC, Red Blood Cell; WBC, White Blood Cell; ICU, Intensive Care Unit; IQR, Interquartile Range; IMCI, Integrated Management of Childhood Illnesses.

Treatment variables often occur in linked clinical pathways; therefore, they are presented for descriptive context only and are not interpreted as independent outcomes.

Supplemental Table 5: P-values for Heterogeneity of EVD68 Effect by Year of Admission

Outcome Measure	P-value for Heterogeneity (Risk Difference)	P-value for Heterogeneity (Risk Ratio)
IMCI Pneumonia	0.426	0.28
Wheeze	0.388	0.239
ICU Admission	0.787	0.91
Oxygen Therapy	0.84	0.755

Abbreviations: IMCI, Integrated Management of Childhood Illnesses; ICU, Intensive Care Unit.

Supplementary Figures

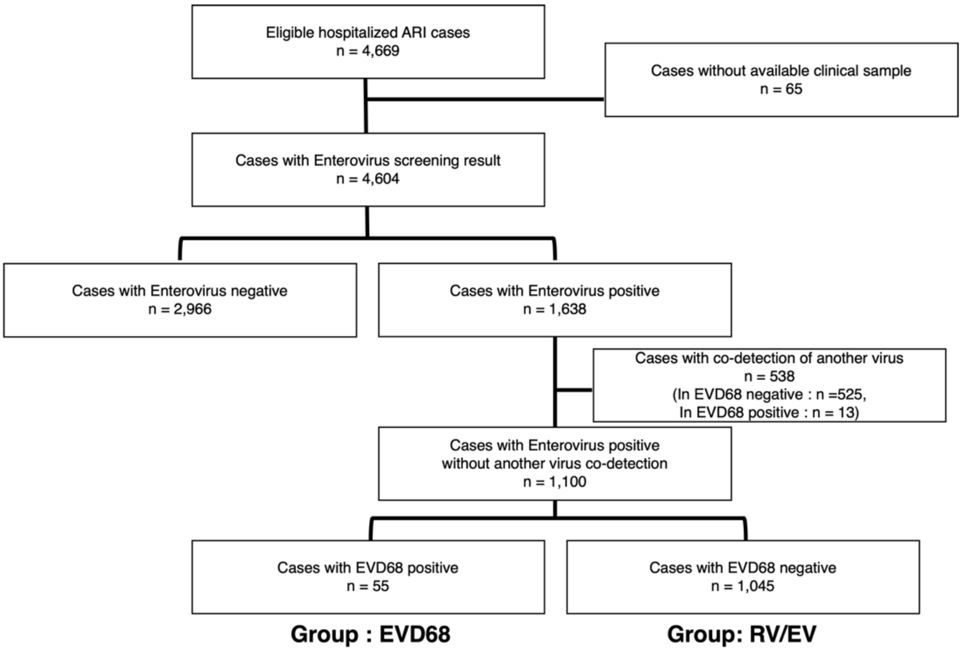

Supplementary Figure 1. Flowchart of participant enrollment and virus detection.

The diagram illustrates the number of hospitalized ARI cases, screening results for enteroviruses, and final analytic groups. The EVD68-negative group is referred to as RV/EV, as the screening PCR detects both rhinoviruses and enteroviruses.

Abbreviations: EVD68, Enterovirus D68; RV/EV, rhinoviruses/enteroviruses.

(a) EVD68

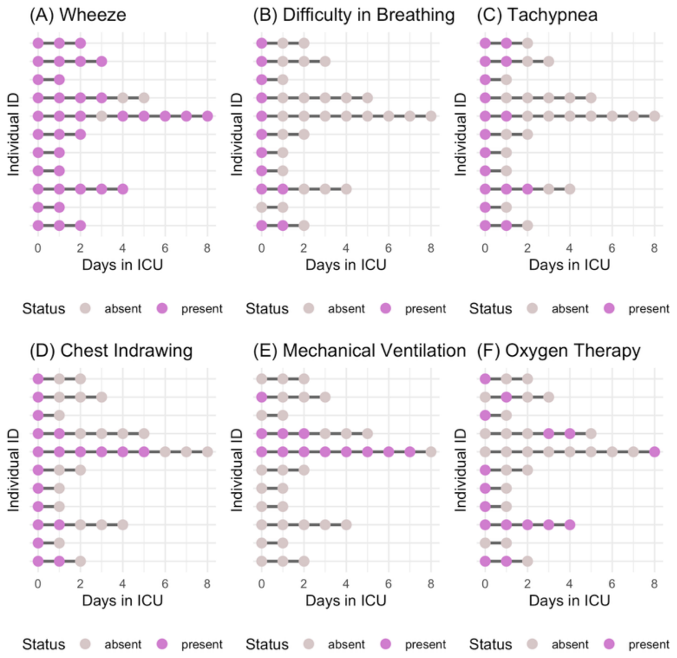

(b) RV/EV

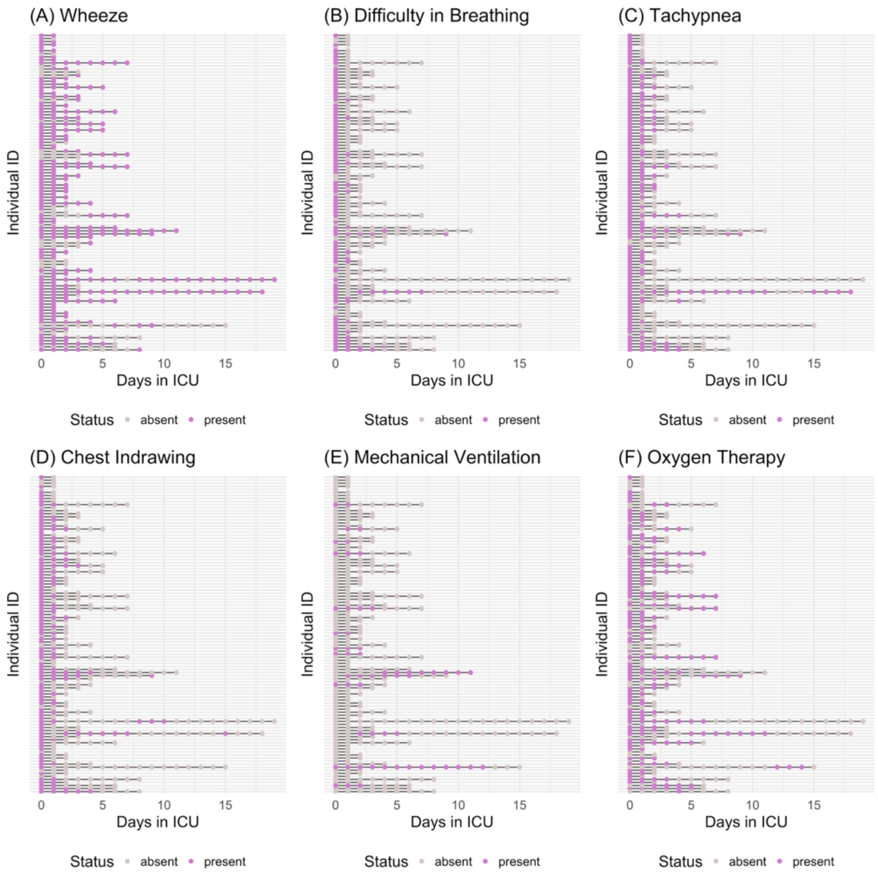

Supplementary Figure 2. Individual-level ICU timelines for EVD68 (panel a) and RV/EV (panel b). Rows are patient IDs and columns are days since ICU admission. For each day, magenta dots indicate presence and gray dots absence of (A) wheeze, (B) difficulty breathing, (C) tachypnea, (D) chest indrawing, (E) mechanical ventilation, or (F) oxygen therapy, visualizing within-ICU progression at the patient level.

Abbreviations: ICU, Intensive Care Unit; EVD68, Enterovirus D68; RV/EV, rhinoviruses/enteroviruses.

References

1. Kiang D, Kalra I, Yagi S, et al. Assay for 5′ Noncoding Region Analysis of All Human Rhinovirus Prototype Strains. *Journal of Clinical Microbiology*. 2008;46(11):3736-3745. doi:10.1128/JCM.00674-08

2. Kiang D, Yagi S, Kantardjieff KA, Kim EJ, Louie JK, Schnurr DP. Molecular characterization of a variant rhinovirus from an outbreak associated with uncommonly high mortality. *Journal of Clinical Virology*. 2007;38(3):227-237. doi:10.1016/j.jcv.2006.12.016

3. Yoshida LM, Suzuki M, Yamamoto T, et al. Viral pathogens associated with acute respiratory infections in central Vietnamese children. *Pediatr Infect Dis J*. 2010;29(1):75-77. doi:10.1097/INF.0b013e3181af61e9

4. Organization WH. Global guidance for conducting acute flaccid paralysis (AFP) surveillance in the context of poliovirus eradication. 2024:ix, 104 p.

Alt Text

Supplementary Figure 1. Figure shows a flowchart of hospitalized acute respiratory infection cases, the number tested for enteroviruses, and the enterovirus-positive cases included in the analysis, resulting in EVD68 and RV/EV groups.

Supplementary Figure 2. Individual ICU timelines for patients with EVD68 and RV/EV infections, showing daily respiratory symptoms and respiratory support levels from the date of admission.
